# Human Transthyretin with a Tailored Gd(III) Complex as a High-Relaxivity MRI Contrast Agent

**DOI:** 10.1021/acs.inorgchem.6c01347

**Published:** 2026-04-21

**Authors:** Jlenia Bindi, Adam Kubrak, Yiqing Lei, Megan Kaster, Linda Cerofolini, Enrico Ravera, Thomas J. Meade, Marco Fragai, Giacomo Parigi

**Affiliations:** † Department of Chemistry “Ugo Schiff” and Magnetic Resonance Center (CERM), 9300 University of Florence, Sesto Fiorentino 50019, Italy; ‡ 524266 Consorzio Interuniversitario Risonanze Magnetiche Metallo Proteine (CIRMMP), via Sacconi 6, Sesto Fiorentino 50019, Italy; § Department of Chemistry, 3270 Northwestern University, 2145 Sheridan Road, Evanston, Illinois 60208, United States; ∥ Departments of Molecular Bioscience, Neurobiology, and Radiology, Northwestern University, 2205 Tech Dr, Evanston, Illinois 60208, United States

## Abstract

Protein-based biomaterials are attractive platforms for medical applications, as they can combine drug delivery, targeting, and diagnostic imaging. In this work, human transthyretin (TTR), a 55 kDa homotetrameric plasma protein with drug-carrier potential, was functionalized with the paramagnetic complex **Gd-C4-IA** to generate a protein-based MRI contrast agent. The gadolinium­(III) complex features a DOTA-like macrocyclic scaffold with a propionic carboxyamide arm for fast water exchange and an iodoacetamide linker for covalent attachment to the protein cysteine residue. Successful conjugation was confirmed via ^1^H NMR paramagnetic broadening and ICP–MS. Analysis of the ^1^H nuclear magnetic relaxation dispersion (NMRD) profiles at 25 and 37 °C indicated a substantial enhancement in relaxivity relative to that of the free complex. Quantitative analysis reveals that the relaxation mechanism is dominated by nanosecond reorientation times, optimized for clinical magnetic fields, though partially averaged by fast local dynamics of the paramagnetic tag. The combination of fast water exchange, favorable rotational dynamics, and multivalent Gd­(III) loading accounts for the remarkable relaxivity gain. Given the physiological role of TTR, this conjugate represents a promising biocompatible platform for combined diagnostic and drug delivery payloads. Furthermore, these data underscore the potential of **Gd-C4-IA** as a highly efficient paramagnetic tag for functionalizing protein-based biomaterials.

## Graphical abstract

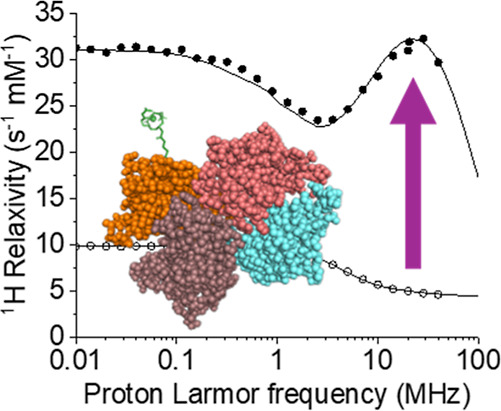

## Introduction

Protein-based biomaterials have been proposed as drug carriers able to ensure extended drug half-lives and efficient targeting to improve their therapeutic efficacy.^1,2^ The conjugation of small drugs to proteins can in fact decrease renal excretion, thus increasing their half-lives,^3^ and the presence of specific recognition of receptors can provide efficient targeting. Proteins also represent an attractive platform for magnetic resonance imaging (MRI) because, when conjugated to Gd­(III) complexes, they can serve as MRI contrast agents with the additional advantage of being able to carry a high Gd­(III) payload and possessing an improved efficacy at clinically relevant field strengths. The first example of a protein used as a carrier of either drugs^4,5^ or contrast agents^6^ is human serum albumin.

The administration of MRI contrast agents can be of great importance for medical diagnosis because they enhance tissue contrast in MR images by increasing the longitudinal relaxation rates, *R*_1_, of nearby water protons. Clinically used MRI contrast agents are small paramagnetic Gd­(III) complexes containing one or two exchanging water molecules coordinated to the metal ion. Their efficiency is determined by the relaxivity, defined as the enhancement in the longitudinal water proton relaxation rate due to a Gd­(III) concentration of 0.001 mol/dm.^3^ An effective way to increase relaxivity is by slowing down the reorientation mobility of the complex, as it can be achieved through the attachment to biomolecules. An increased efficiency is also beneficial to reduce the administered dose of Gd­(III) complexes, thus reducing the risks associated with their accumulation in the tissues of the patients,^7,8^ and to nephrogenic systemic fibrosis in patients with impaired renal clearance.^9–11^

Gd­(III) DOTA-like or DTPA-like complexes with an electrophilic group for conjugation to nucleophilic groups of macromolecules,^12^ were thus proposed as paramagnetic tags to attach to protein-based biomaterials. The ease of genetically engineering protein polymers at multiple backbone sites allows for the attachment of multiple paramagnetic tags per protein,^13,14^ resulting in carriers with very high relaxivity per particle. A Gd­(III) DOTA derivative was, for instance, covalently attached to asparaginase, a biological drug in clinical use against leukemia, through a carboxylate group activated with the ester and the primary amine of the N-terminus and lysine residues via amide bond formation.^15^ A gadolinium triacetic monoamide DOTA derivative with a methanethiosulfonate anchor group was also shown to form disulfide bonds with albumin in its native and reduced forms and with thiolated silica particles.^16^ The relaxivity of this system remains suboptimal, despite the large increase in the reorientation correlation times because it is limited by the slow exchange time of the water molecule coordinated to the gadolinium­(III) ion.

The paramagnetic complex **Gd-C4-IA** ^17^ (Scheme 1), comprising a gadolinium­(III) ion, a DOTA-like ligand, and a linker for the binding to the –SH group of the cysteine amino acid of the protein, has been shown to possess more favorable exchange times, thanks to an increased length of the carboxyamide arm. In this derivative, in fact, the increase from acetic to propionic of the amide pendant arm boosts the water exchange rate by almost 2 orders of magnitude with respect to the monoacetoamide DOTA derivatives.^18^ Their possibility of providing integrated platforms was previously shown through the attachment to AaLS-13 and OP cages, where high relaxivity and signal amplification was observed.^17^

**Scheme 1:**
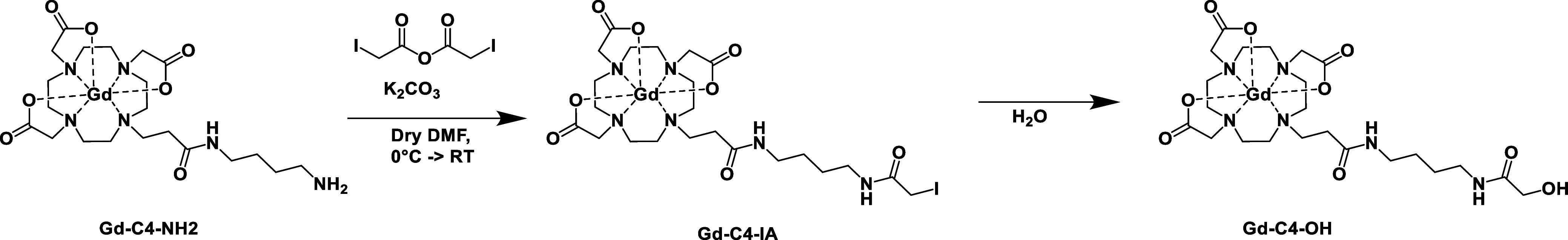
Synthetic Route to the Bioconjugation-Ready Gadolinium Complex **Gd-C4-IA** and Formation of Its Hydrolysis Byproduct^a^

a The precursor **Gd-C4-NH**_**2**_, bearing a terminal primary amine on the C4 linker, was reacted with iodoacetic anhydride in dry DMF in the presence of K_2_CO_3_ (0 °C → room temperature) to afford the iodoacetamide-functionalized complex **Gd-C4-IA**, which serves as a thiol-reactive handle for bioconjugation. Under aqueous conditions, the iodoacetamide moiety undergoes hydrolysis to form the corresponding hydroxyacetamide derivative **Gd-C4-OH**.^17^ The scheme illustrates both the formation of the bioconjugation intermediate and the competing hydrolytic pathway observed during purification.^17^

Human transthyretin (TTR hereafter), a homotetrameric protein with a total molecular mass of 55 kDa, has also been considered as a drug carrier. This protein is present in blood plasma and cerebrospinal fluid, where it carries the holo-retinol binding protein and the thyroxine T4 hormone. TTR has already been identified as a possible carrier protein for the delivery of cytotoxic drugs to cancer cells.^19–22^ This would allow for its use as a protein-drug conjugate in cancer therapy. The high affinity of the drug with the protein can in fact allow for the delivery of hydrophobic cytotoxic drugs that would otherwise result in poor solubility.

TTR has a single cysteine residue located in position 10, close to the N-terminal domain of the protein. The protein TTR is here labeled with the paramagnetic complex **Gd-C4-IA**. **Gd-C4-IA** was attached by conjugation by the alkylation of the cysteine thiols with the iodoacetamide group of **Gd-C4-IA**.

## Methods

### Synthesis of **Gd-C4-IA**

The Gd­(III) complex **Gd-C4-IA** was synthesized as previously described by Kaster et al.^17^ (Scheme 1). Briefly, **Gd-C4-NH**_**2**_ was purified by semipreparative HPLC using an Agilent PrepStar 218 system equipped with an Agilent 1260 Infinity diode-array detector and a Waters Atlantis T3 column (100 Å, 10 μm, 19 × 250 mm). The purity of **Gd-C4-NH**_**2**_ was confirmed by analytical HPLC–MS using an Agilent 1260 Infinity II HPLC system coupled to an Agilent 6120 quadrupole mass spectrometer, with separation performed on a Waters Atlantis T3 column (100 Å, 5 μm, and 4.6 × 250 mm).

All HPLC purifications and analytical measurements were carried out using ultrapure water (18.2 MΩ·cm) obtained from a Millipore Synergy UV water purification system and HPLC-grade acetonitrile (Fisher Scientific) under neutral conditions.

The purified **Gd-C4-NH**_**2**_ complex was reacted with 3 equiv of iodoacetic anhydride (Sigma-Aldrich) in the presence of 3 equiv of K_2_CO_3_ (Fisher Scientific) at 0 °C under an inert atmosphere, and the reaction mixture was allowed to warm to room temperature and stirred overnight. Reaction progress was monitored by electrospray ionization mass spectrometry (ESI-MS) using a Bruker amaZon SL mass spectrometer. After completion, the solvent was removed under reduced pressure, and the crude product was resuspended in water and rapidly purified by reverse-phase HPLC (RP-HPLC). The purified **Gd-C4-IA** was lyophilized to afford a white powder.

### Nuclear Magnetic Relaxation Dispersion (NMRD) Measurements

^1^H nuclear magnetic relaxation dispersion (NMRD) profiles were recorded with a SPINMASTER2000 fast field cycling relaxometer (Stelar, Mede (PV), Italy) operating in the 0.01–40 MHz ^1^H Larmor frequency range. The measurements are affected by an error of about ±1%, as obtained in the field cycling experiment from the fit to a monoexponential decay/recovery of the magnetization.

### Sample Preparation

TTR was expressed using *E. coli* BL21­(DE3) Codon Plus RIPL cells transformed with the pET28a plasmid encoding human TTR. Antibiotics used were chloramphenicol and kanamycin; the cells were grown at 37 °C, and expression was induced with 1 mM IPTG. After purification, the unlabeled TTR was conjugated to the **Gd-C4-IA** complex. The reaction was conducted under an inert atmosphere, maintained using a glovebox, protected from light and at room temperature. An excess (4.1 equivalents) of **Gd-C4-IA** was directly added to the buffer containing TTR (25 mM Tris, pH 7.6, 200 mM NaCl, 5 mM EDTA) and left overnight under mild magnetic stirring. The protein concentration for the reaction was approximately 10 mg/mL. The excess unreacted **Gd-C4-IA** was then removed with a PD-10 desalting column. The sample was purified using Superdex 75pg, pre-equilibrated with 25 mM Tris (pH 7.6), 200 mM NaCl, and 5 mM EDTA. 1D ^1^H NMR spectra were acquired and compared to those of the unconjugated protein, showing the disappearance of several protein signals due to paramagnetic broadening. ICP–MS measurements indicated that the concentration of the Gd­(III) ions was 40 μM, with a concentration of the protein monomers of 200 μM. These values reflect a stoichiometry of nearly one gadolinium­(III) ion per protein tetramer.

## Results and Discussion

### Preparation of Gd-Labeled TTR

The gadolinium-based contrast agent **Gd-C4-IA** was synthesized, as previously described.^17^ The intermediate **Gd-C4-NH**_**2**_ was purified by reverse-phase high-performance liquid chromatography (RP-HPLC) and displayed a single peak at a 12.5 min retention time by analytical HPLC, consistent with previously reported values (Figure 1A). The purified **Gd-C4-NH**_**2**_ intermediate was subsequently used to generate the final bioconjugation-ready complex **Gd-C4-IA**.

**Figure 1:**
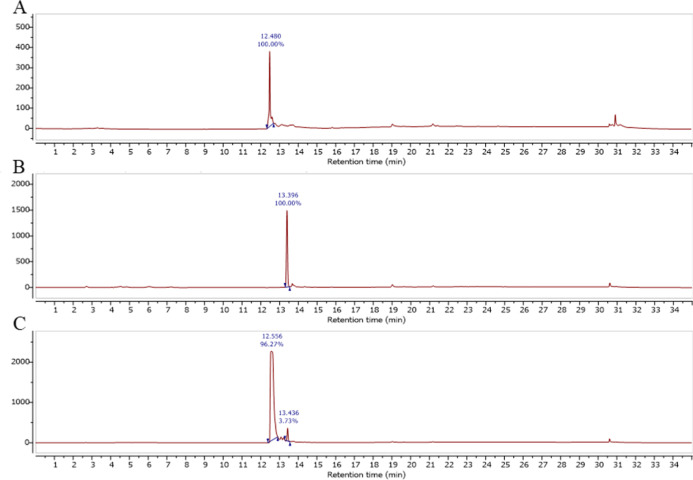
Analytical HPLC chromatograms of the Gd complexes. (A) HPLC trace of **Gd-C4-NH**_**2**_ showing a single peak with a retention time of 12.5 min. (B) HPLC trace of **Gd-C4-IA** showing a peak at 13.4 min. (C) HPLC trace of **Gd-C4-OH** showing a peak at 12.6 min; the additional peak at 13.4 min corresponds to **Gd-C4-IA**.

The identity of **Gd-C4-IA** was confirmed by analytical HPLC, which showed a single peak with a 13.4 min retention time, comparable to the previously reported 13.5 min retention time obtained using the same HPLC method (Figure 1B).^17^ Detailed characterization of the complex, including high-resolution mass spectrometry (HRMS), has been previously reported.^17^

During purification, a hydrolyzed byproduct, **Gd-C4-OH**, was isolated at 11.5 min retention time by semipreparative HPLC. When analyzed under analytical HPLC conditions, this species exhibited a 12.6 min retention time (Figure 1C). Notably, **Gd-C4-OH** elutes at essentially the same retention time as **Gd-C4-NH**_**2**_ under analytical HPLC conditions.

### Relaxation Profiles of Diamagnetic TTR

Water proton relaxation rates can be enhanced in the presence of diamagnetic proteins because the interaction with the protein slows water reorientation and thus increases the correlation time of the magnetic proton–proton dipole–dipole interactions. The ^1^H NMRD profiles report the field-dependent relaxation rates of water protons.^23–26^ Their field dependence is determined by the correlation times modulating the proton–proton dipole–dipole interactions.

^1^H NMRD profiles were obtained for a 1.2 mM water solution of the free wild-type TTR by measuring the water proton relaxation rates, *R*_1_, as a function of the applied magnetic field.^19^ The profiles acquired at 25 and 37 °C are shown in Figure 2. Multiple correlation times must be considered to account for the many motional processes of the different water protons interacting with the protein. These correlation times are the fastest between the proton lifetimes and the reorientation times, comprising both the overall protein tumbling and faster protein local dynamics.

**Figure 2:**
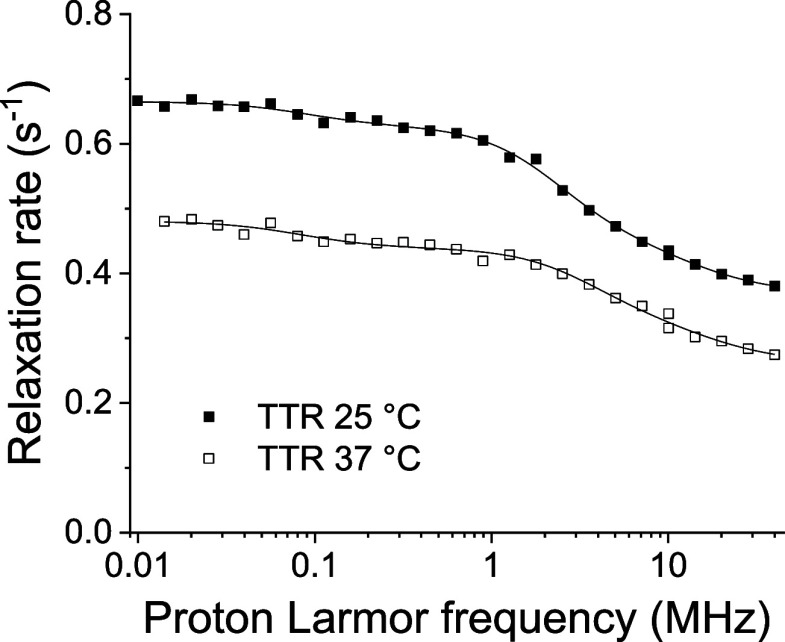
^1^H NMRD profiles of a water solution of TTR (1.2 mM monomeric protein concentration) at 25 and 37 °C.

The profiles could be fitted according to the model-free approach^24,25,27^

$$R_{1}=\alpha +\beta \sum_{i}^{N}c_{i}\left(\frac{\tau_{i}}{1+\omega_{I}^{2}\tau_{i}^{2}}+\frac{4\tau_{i}}{1+4\omega_{I}^{2}\tau_{i}^{2}}\right)$$

where multiple relaxation contributions arising from different correlation times, τ_*i*_, are considered, with *c*_*i*_ as weight coefficients summing to 1 (ω_I_ is the nuclear Larmor frequency). In order to reduce the covariance among the many parameters, the parameters β and the weight coefficients were constrained to be the same for the two temperatures.

Three correlation times were needed for the fit of the profiles. The longest correlation time, of the order of a thousand nanoseconds, has very low (≪1%) weight coefficients, and it likely corresponds to contributions from aggregated forms of the protein that formed at this high concentration. The shortest correlation time is of the order of nanoseconds, thus reporting on the internal local protein mobility and/or the lifetime of proton exchange processes. The intermediate correlation time, with a weight coefficient of ca. 0.30 ± 0.01, amounts to 36 ± 4 and 23 ± 3 ns at 25 and 37 °C, respectively, hence corresponding to the overall reorientation time of the protein. The overall tumbling time expected for tetrameric TTR was in fact calculated with HydroNMR^28^ to amount to 29 and 22 ns at 25 and 37 °C, respectively.

### Relaxation Profiles of the Paramagnetic Complex

A much larger increase in water proton relaxation rates can be achieved in the presence of paramagnetic complexes due to the magnetic dipole–dipole interactions between water protons and unpaired electrons present in the paramagnetic metal. Gd­(III) is an excellent metal to increase water proton relaxation rates because of the high spin state (*S* = 7/2) and long electronic relaxation time τ_e_. The relaxivity of the Gd­(III) complex is determined from the difference between the relaxation rates measured for a water solution of the complex and those of the buffer solution, normalized to a 1 mM Gd­(III) concentration. Two terms can contribute to the relaxivity *r*_1_:

1 $$r_{1}=\frac{10^{-3}q}{55.5}{\left(R_{1M}^{-1}+\tau_{M}\right)}^{-1}+R_{1}^{out}$$

i.e., an inner-sphere contribution arising from *q* exchangeable water molecules coordinated to the Gd­(III) ion with a mean residence time τ_M_ and with relaxation rate *R*_1M_, and an outer-sphere contribution arising from the dipole–dipole interaction between the unpaired electron(s) and the protons of freely diffusing water molecules. The relaxation rate of the inner-sphere water protons, *R*_1M_, is described by the Solomon-Bloembergen-Morgan (SBM) theory

2 $$R_{1M}=\frac{2}{15}{\left(\frac{\mu_{0}}{4\pi }\frac{\gamma_{I}{g\mu }_{B}}{r^{3}}\right)}^{2}S\left(S+1\right)\left[\frac{7\tau_{c}}{1+\omega_{s}^{2}\tau_{c}^{2}}+\frac{3\tau_{c}}{1+\omega_{I}^{2}\tau_{c}^{2}}\right]$$

3 $$\tau_{c}^{-1}=\tau_{r}^{-1}+\tau_{e}^{-1}+\tau_{M}^{-1}$$

4 $$\tau_{e}^{-1}=\frac{2\Delta_{t}^{2}}{50}\left[4S\left(S+1\right)-3\right]\left[\frac{\tau_{v}}{1+{\omega_{S}^{2}\tau }_{v}^{2}}+\frac{{4\tau }_{v}}{1+{{4\omega }_{S}^{2}\tau }_{v}^{2}}\right]$$

where *r* is the distance between the protons of the coordinated water molecule and the paramagnetic metal ion, ω_S_ = 658.2ω_I_ is the electron Larmor frequency, τ_R_ is the reorientation time, Δ_*t*_^2^ is the mean squared fluctuation of the zero-field splitting, and τ_v_ is the correlation time for the instantaneous distortions of the coordination polyhedron of the paramagnetic metal. These equations were derived under a number of approximations,^29^ among which is the absence of static zero-field splitting (ZFS).

According to the hard-sphere spherical model,^30,31^ the outer-sphere contribution, provided by water molecules freely diffusing around the paramagnetic moiety up to a distance of closest approach *d*, is given by

$$\begin{array}{c} R_{1}^{out}=\frac{32\pi }{405}{\left(\frac{\mu_{0}}{4\pi }\right)}^{2}\frac{{N_{A}\left(\gamma_{I}\mu_{B}g_{e}\right)}^{2}S\left(S+1\right)}{dD}\\ \left[7J\left(\omega_{S},\tau_{D},\tau_{e}\right)+3J\left(\omega_{I},\tau_{D},\tau_{e}\right)\right] \end{array}$$ 5

where *N*_A_ is the Avogadro’s constant, *D* is the water diffusion coefficient, τ_*D*_ = *d* ^2^/*D*, and

6 $$J\left(\omega ,\tau_{D},\tau_{e}\right)=Re\left[\frac{1+\frac{\Omega^{1/2}}{4}}{1+\Omega^{1/2}+\frac{4\Omega }{9}+\frac{\Omega^{3/2}}{9}}\right]$$

with

7 $$\Omega =i\omega \tau_{D}+\tau_{D}/\tau_{e}$$

Figure 3 shows the relaxivity profiles at 25 and 37 °C measured for a 1 mM water solution of the **Gd-C4-OH**, where the iodine was substituted by a hydroxy group. The profiles, in excellent agreement with previous measurements,^17^ indicate the presence of one fast-exchanging water molecule regularly coordinated to the Gd­(III) ion. The profiles were fitted to eqs 1–7, and the best fit parameters are reported in Table 1. The decrease in relaxivity observed with increasing temperature across the whole range of frequencies indicates that τ_M_ < *R*_1M_^–1^ (fast exchange regime, see eq 1); so the water lifetime τ_M_ is in the range of 10^–8^–10^–7^ s. The occurrence of exchange in this range of times ensures that τ_M_ is basically not affecting the relaxation mechanism and thus the measured relaxation profiles. In the fit, the values of τ_M_ were thus fixed to ca. 10^–8^ s, in agreement with previous measurements performed on analogous derivatives.^18^

**Figure 3:**
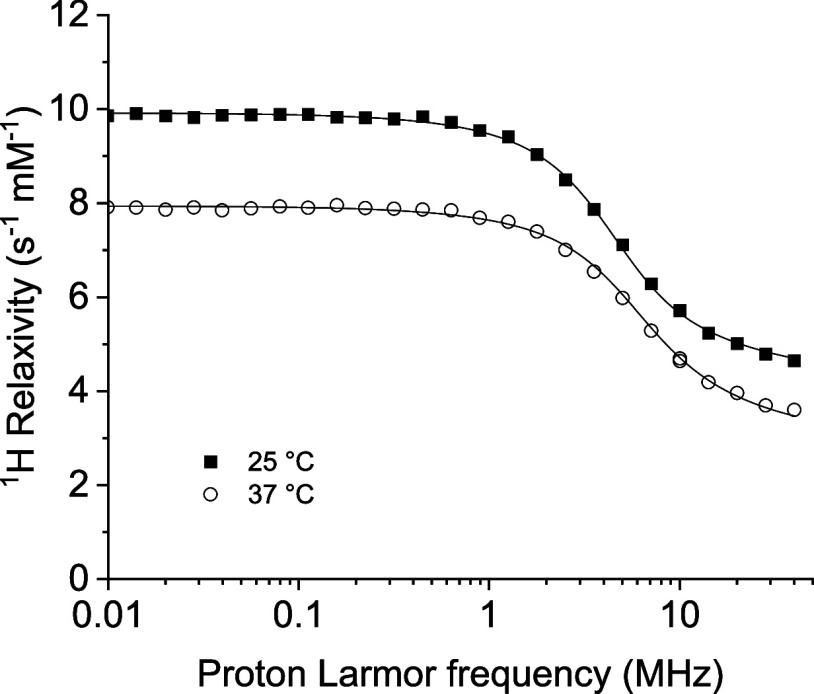
^1^H relaxivity profiles of **Gd-C4-OH** at 25 and 37 °C. Solid lines are the best fit profiles obtained with eqs 1–7.

**Table 1:** Best Fit Parameters of the ^1^H Relaxivity Profiles

	Gd-C4-OH		Gd-conjugated TTR		
	25 °C	37 °C	25 °C	37 °C	
*r* ^a^	3.05				Å
*q* ^a^	1				
Δ_*t*_	0.026 ± 0.002		0.0085 ± 0.0002		cm^–1^
τ_v_	23 ± 4	21 ± 4	25 ± 2	22 ± 2	×10^–12^ s
τ_R_	0.081 ± 0.002	0.054 ± 0.002	2.2 ± 0.3	1.8 ± 0.3	×10^–9^ s
τ_M_ ^a^	12	7	12	7	×10^–9^ s
*S* ^2^			0.51 ± 0.04		
τ_l_			1.2 ± 0.3	0.73 ± 0.3	×10^–10^ s
ZFS			0.018 ± 0.002		cm^–1^
θ			50 ± 3		degrees
*d*	4.0 ± 0.1		4.0^a^		Å
*D* ^a^	2.4	3.1	2.4	3.1	×10^–9^ m^2^/s

a Fixed values.

The correlation time modulating the dipole–dipole interaction is the shortest between the electron relaxation time, the water proton lifetime, and the reorientation time (see eq 3). Reorientation times smaller than 100 ps, as also obtained for this complex, determine the value of τ_c_ at clinically relevant field strengths (20–150 MHz) and limit the relaxivity. In fact, the maximum relaxivity at these fields is experienced when τ_c_^opt^ is equal to ω_I_^–1^, i.e., to a few ns. Tethering Gd­(III) complexes to macromolecules can increase the correlation time τ_c_ by slowing molecular reorientation, thus increasing the relaxivity.

### Relaxation Profiles of the Paramagnetic-Labeled TTR

The NMRD profiles of the water solution of Gd-conjugated TTR were collected at 25 and 37 °C. The concentration of the gadolinium­(III) ions was 0.04 mM, as determined from ICP–MS measurements. The relaxivity values, obtained from the difference between the relaxation rates measured from the paramagnetic and the diamagnetic samples and scaled to 1 mM gadolinium­(III) concentration, are shown in Figure 4.

**Figure 4:**
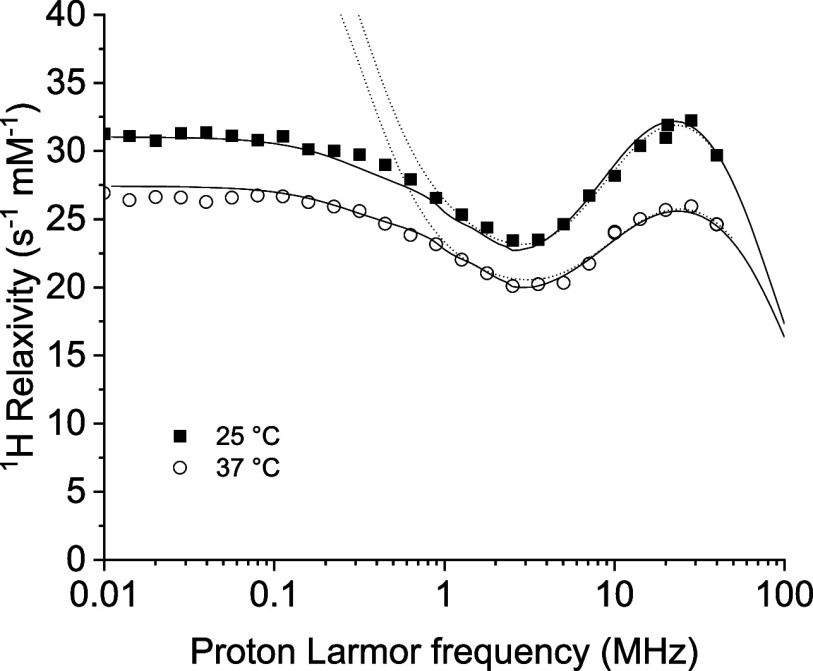
^1^H relaxivity profiles of Gd-conjugated TTR at 25 and 37 °C. Solid lines are the best fit profiles obtained with the Florence NMRD program, and dashed lines are calculated with the SBM model.

Notably, the relaxivity at about 1 T is remarkably high, exceeding that of clinically used contrast agents with *q* = 1 by more than 5 times. As is clear from the presence of the high field peak, the increase in the reorientation time is primarily responsible for this relaxivity enhancement. The profiles were subsequently analyzed using established models to shed light quantitatively on the origin of this enhancement.

The profiles could not be fitted with the SBM model, even including contributions from fast local mobility (with correlation time τ_l_) through a Lipari-Szabo model-free approach.^32,33^ This is due to the presence of ZFS, which is expected to affect the energy of the electron spin states and thus their transition probabilities.^29^ The data were thus fitted using the modified Florence NMRD program.^34–36^ The best fit parameters are reported in Table 1, and the corresponding profiles are shown in Figure 4 as solid lines. The dashed lines in Figure 4 show the relaxivity profiles calculated with the same parameters and using the SBM model.

The temperature dependence indicates that the water molecule coordinated to the Gd­(III) ion is still in a fast exchange. The fit shows that reorientation times τ_R_ of the order of nanoseconds are needed to reproduce the profiles. These times are 1 order of magnitude smaller than the tumbling times of the tetrameric protein (see above), which implies that the dipole–dipole interactions between the unpaired electrons of the gadolinium­(III) ions and the water protons are completely averaged out by internal dynamics. These times are, however, in the optimal range for achieving a maximum relaxivity at the imaging fields.

In order to reproduce the profiles, a second, faster reorientation time, τ_l_, was, however, needed, defining a further correlation time

8 $$\tau_{c2}^{-1}=\tau_{l}^{-1}+\tau_{r}^{-1}+\tau_{e}^{-1}+\tau_{M}^{-1}$$

introduced to modulate the dipole–dipole interaction according to the Liparis Szabo model-free approach. The *S* ^2^ order parameter, providing the weight of the slower correlation time, resulted of ca. 0.5, indicating that about half of the relaxation process is modulated by a faster local mobility occurring on a time scale of ca. 100 ps (τ_l_), i.e., of the reorientation time of the paramagnetic tag. This indicates that the high flexibility of the Gd­(III) tag allows for extensive reorientation of the Gd­(III) complex.

Concerns regarding the kinetic instability of gadolinium chelates—particularly transmetalation with endogenous metal ions such as Zn^2+^ leading to the release of free, toxic Gd^3+^—have historically been associated with early linear chelators based on DTPA. These concerns ultimately led to regulatory restrictions and the withdrawal of several linear Gd-based contrast agents by the European Medicines Agency in 2017. In contrast, macrocyclic gadolinium chelators exhibit substantially greater kinetic inertness and resistance to transmetalation due to their preorganized ligand architecture. Reported dissociation rate constants for macrocyclic systems (*k*_obs_ ≈ 10^–7^ s^–1^) are several orders of magnitude lower than those of linear chelators (*k*_obs_ ≈ 10^–4^ s^–1^).^37,38^ The **Gd-C4-IA** complex described here is derived from a clinically validated macrocyclic scaffold (ProHance), which has an established safety profile.

Macrocyclic Gd chelators, including ProHance and **Gd-C4-IA**, also display high thermodynamic stability (log *K* ≈ 24). Importantly, under physiological conditions, release of free Gd^3+^ in buffer or human serum is typically below the limit of detection over extended timeframes (e.g., up to 15 days), indicating strong resistance to transmetalation and excellent in situ stability.^37,39,40^

Table 2 summarizes the relaxivity values at various magnetic fields for previously reported Gd-tagged proteins, along with some noncovalent protein adducts and chimeric proteins. Table 3 presents the reorientation and water chemical exchange parameters obtained from the best-fit analysis of the NMRD profiles at 37 °C for proteins covalently labeled with *q* = 1 gadolinium­(III) tags. In most cases, relaxivity values in the range of 25–30 s^–1^ mM^–1^ are achieved when proteins are conjugated to **Gd-C4-IA**. This relaxivity is primarily limited by a squared order parameter for the nanosecond reorientation time, which remains significantly lower than 1 when the **Gd-C4-IA** tag is attached to TTR, AaLS-13, or OP (either internally or externally to the protein cage). Notably, when three **Gd-C4-IA** tags are attached to the interior of the OP protein cage, the relaxivity increases substantially due to the enhanced rigidity of the tag, leading to a fast correlation time τ_l_ approaching 1 ns. Conversely, for the **Gd-DOTA-NHS**-ester, the relaxivity is limited by the slow exchange of the coordinated water molecule.

**Table 2:** Literature Reported Gd­(III) Protein Conjugates and Their Relaxivity Measured at Various Field Strengths

		relaxivity (s^–1^ mM^–1^)				
tag	protein	0.5T	1.4T	7T		ref
**Gd-C4-IA**	TTR	26				this report
**Gd-C4-IA**	AaLS-13	27	18.3	8.0		^17^
**Gd-C4-IA**	OP (1_ext_)	16	11.2	4.9		^17^
**Gd-C4-IA**	OP (1_int_)	29	15.0	4.6		^17^
**Gd-C4-IA**	OP (3_int_)	39	15.9	5.3		^17^
**Gd-DOTA-NHS**-ester	ANSII	35				^15^
Gd-DTPA	IgG	15				^41^
Gd-DTPA	BSA	20				^41^
Gd-L1A	cell surface protein thiol			2.0		^42^
Gd-L1B	cell surface protein thiol			2.3		^42^
Gd-DO3A-SA-biot	avidin	17.2			non covalent	^43^
MS-325	HSA	42			non covalent	^33^
GdL1	HSA	68			non covalent	^44^
Gd^III^-H_4_L1	HSA	52			non covalent	^45^
ProCA32s			34 (1.5T)		chimeric protein	^46^

**Table 3:** Best Fit Data Reported from the Analysis of the NMRD Profiles at 37 °C for Gd-Tagged Proteins with One Water Molecule Coordinated to the Paramagnetic Metal Ion

tag	protein	τ_R_ (ns)	*S* ^2^	τ_l_ (ns)	τ_Μ_ (ns)	ref
**Gd-C4-IA**	TTR	1.8	0.51	0.07	7	this report
**Gd-C4-IA**	AaLS-13	3.5	0.28	0.2	100	^17^
**Gd-C4-IA**	OP (1_ext_)	1.1	0.36	0.03	70	^17^
**Gd-C4-IA**	OP (1_int_)	3.3	0.36	0.04	70	^17^
**Gd-C4-IA**	OP (3_int_)	3.3	0.46	0.5	70	^17^
**Gd-DOTA-NHS**-ester	ANSII	3.4	0.71	0.08	230	^15^

## Conclusions

The Gd­(III) complex **Gd-C4-IA** has been shown to provide an easy and effective paramagnetic tag to increase the water proton relaxivity through its attachment to target proteins. The complex was designed using the macrocyclic cyclen scaffold of clinically approved contrast agents in order to exhibit good thermodynamics and kinetic stability. The optimal fast water exchange rate circumvents the bottleneck of slow water exchange that often plagues macromolecular Gd­(III) conjugates, and the presence of a pendant linker for the binding to the cysteine amino acids of a protein allows its easy conjugation to a variety of biomolecular platforms.

Given the natural role of TTR as a carrier for thyroxine and retinol-binding protein,^47–49^ and its established potential for transporting cytotoxic drugs,^20^ protein conjugation of **Gd-C4-IA** provides a robust molecular tool for the development of targeted MRI contrast agents and the monitoring of protein-based drug delivery systems.
